# Evaporation Mechanisms and Variability Prediction of Concrete in Dynamic Urban Road Environments

**DOI:** 10.3390/ma19214599

**Published:** 2026-10-30

**Authors:** Qiang Xu, Wanpeng Yang, Yanfeng Zhao, Meiqian Wang, Qingwei Li, Wei Xu

**Affiliations:** 1 Faculty of Civil Engineering and Mechanics, Kunming University of Science and Technology, Kunming 650500, China; structlab@126.com (Q.X.);; 2 School of Architecture and Engineering, Yunnan Land and Resources Vocational College, Kunming 652501, China; 3 Yunnan Aerospace Engineering Geophysical Exploration and Detection Co., Ltd., Kunming 650217, China; 4 Intelligent Infrastructure Operation and Maintenance Technology Innovation Team of Yunnan Provincial Department of Education, Kunming University of Science and Technology, Kunming 650500, China; 5 Honghe Highway Bureau, Honghe 661199, China

**Keywords:** urban road concrete, dynamic environment, evaporation mechanisms, pore structure, degree of hydration, predictive models

## Abstract

During long-term service, concrete on urban roads is exposed to dynamic coupled environments, including diurnal temperature fluctuations, wind disturbance from traffic, and cyclic vibration from vehicle loads. Its early moisture evaporation behavior differs essentially from that in static atmospheric conditions. Clarifying the evaporation mechanism and evolution rules is a core scientific issue for improving the service durability of pavement concrete. In this study, C30 and C50 concrete mixes widely used in urban road engineering were taken as research objects. A self-developed dynamic environment simulation test bench was adopted, combined with macroscopic evaporation monitoring and microscopic methods including scanning electron microscopy (SEM), mercury intrusion porosimetry (MIP) and thermogravimetric analysis (TGA). The moisture evaporation characteristics of concrete under temperature/wind vibration coupling were systematically studied. The microscopic mechanism of dynamic evaporation was revealed, and a multi-factor coupled evaporation prediction model was established. The results show that a 5 °C temperature rise and 2 m/s wind speed increase raise the early evaporation rate by 32.5% and 28.7% respectively. Vibration at 30 Hz improves surface pore connectivity by 15.3–21.5%. Under dynamic coupled conditions, the 14-day cumulative evaporation of C30 and C50 concrete increases by 41.2% and 35.8% compared with static conditions, accompanied by reduced hydration degree and coarsened pore structure. The proposed model has an average relative error of 6.5% and a coefficient of variation of 7.8%, with significantly higher accuracy than classical static evaporation models. This work can provide a theoretical basis and technical support for mix optimization, early moisture curing improvement and durability enhancement of urban road concrete.

## 1. Introduction

Rapid urbanization has brought dual challenges to urban roads: growing traffic loads and fluctuating environmental conditions. During long-term service, pavement concrete is constantly affected by diurnal temperature changes, wind induced by moving vehicles, and cyclic traffic vibration. Its performance evolution differs essentially from that in static atmospheric environments.

Moisture evaporation is a core physical process governing early concrete performance. It directly affects cement hydration, drying shrinkage, and the development of mechanical properties and durability [1,2,3]. Abnormal evaporation from complex dynamic environments causes rapid surface moisture loss. This leads to insufficient hydration, coarsened pores and surface microcracks, which eventually degrade flexural strength, impermeability and frost resistance, and greatly shorten road service life [4,5,6]. Therefore, clarifying evaporation rules and mechanisms under dynamic urban road conditions is critical for improving pavement concrete durability.

Most existing studies on concrete evaporation are based on static atmospheric environments, and a mature theoretical framework has been formed. Wong et al. [7] stated that temperature and humidity are key evaporation factors, and high temperature with low humidity significantly accelerates moisture loss. Li et al. [8] pointed out that humidity gradient is the main driving force for internal moisture migration, and surface evaporation creates an inward-to-outward humidity difference. These studies lay the classical foundation for concrete evaporation theory, but they all rely on static assumptions. They deviate from actual urban road conditions and cannot guide performance regulation in dynamic scenarios.

In recent years, researchers have gradually focused on the effect of single dynamic factors on concrete evaporation. Guo et al. [9] investigated how temperature fluctuation and wind speed change affect surface evaporation rate, confirming that dynamic conditions alter the surface gas boundary layer and influence evaporation. Cheng et al. [10] found that load vibration changes internal pore structure and regulates moisture migration paths.

However, research gaps still remain. Most studies are limited to independent single-factor effects. The evaporation evolution and synergistic mechanism under combined temperature, wind speed and vibration have not been clarified. The microscopic mechanism mostly stays at a qualitative level, lacking quantitative links between pore evolution and hydration. Meanwhile, most existing prediction models are built for static environments. They exclude road-specific factors like traffic vibration and dynamic wind speed, so their applicability to real urban roads is limited [11,12].

To fill these gaps, this study takes C30 and C50 concrete mixes as research objects. A self-built dynamic environment simulation test bench was used to reproduce the coupling of temperature, wind speed and load vibration. Through evaporation tests and microscopic characterizations, the evaporation characteristics under multi-factor coupling were systematically studied. The internal mechanism of “moisture migration—structure evolution—hydration change” at the microscopic scale was revealed. Finally, a multi-factor prediction model considering temperature, wind speed, vibration frequency, age and water/binder ratio was established and verified. The results can support mix optimization, curing improvement and durability enhancement for urban road concrete.

## 2. Materials and Methods

### 2.1. Specimen Preparation

C30 and C50 concrete mixes, commonly used in urban road engineering, were designed in accordance with the Technical Specification for Construction of Highway Cement Concrete Pavements (JTG/T 3370.1-2021) [13]. P·I 42.5 Portland cement was used, with its chemical composition listed in Table 1. Coarse aggregates were 5–20 mm continuously graded basalt; fine aggregates were medium sand with a fineness modulus of 2.6. Slag powder was added to C50 concrete. The mix proportions are shown in Table 2.

Concrete was mixed in a forced mixer and cast into 100 mm × 100 mm × 400 mm prismatic molds. After compaction on a vibrating table, specimens were kept at room temperature for 24 h before demolding. They were then cured in a standard room (20 ± 2 °C, RH ≥ 95%) to target ages.

A total of 288 specimens were prepared: 168 for C30 and 120 for C50. For all evaporation, microscopic characterization and mechanical tests, three parallel specimens were set for each working condition. The arithmetic average value of qualified parallel samples was taken as the final test index; data with deviation exceeding ±10% from the group average were identified as abnormal outliers and eliminated before averaging.

Specimen numbering follows a unified rule: C/C5 stands for C30/C50 concrete; A represents static control group, AV represents dynamic vibration group; 7/28/60 indicates curing age in days. For example, C30-A28V refers to 28-day C30 concrete under temperature/wind vibration coupling. All specimen codes in the figures and tables follow this logic.

### 2.2. Dynamic Environment Simulation and Evaporation Test

**Figure 1:**
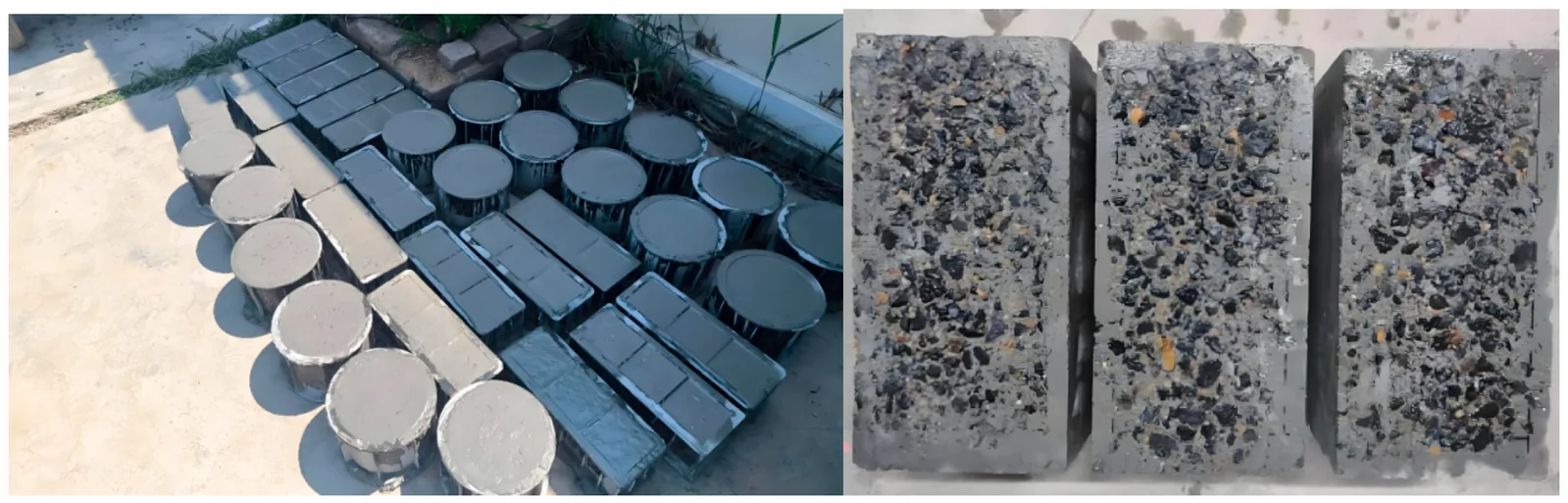
Dynamic environment simulation test bench for urban roads.

A self-developed dynamic environment simulation test bench was used (Figure 1). It consists of an environmental chamber, load vibration system, wind speed control system and temperature/humidity monitoring system. It can realize independent and coupled control of temperature, wind speed and vibration to reproduce real urban road conditions:

(1) Temperature range: −10 to 60 °C, accuracy ±0.5 °C, for simulating diurnal and seasonal temperature changes.
(2) Wind speed range: 0 to 10 m/s, accuracy ±0.2 m/s, for simulating airflow disturbance from vehicles.
(3) Vibration system: frequency 0–50 Hz, amplitude 0–5 mm, contact pressure 0–0.8 MPa, for simulating traffic-induced vibration.
(4) Monitoring system: sensors placed on the surface and at 5 mm, 10 mm, 20 mm depth, recording moisture and temperature every 10 min.

Test variable levels were set as: temperature (20, 25, 30, 35 °C), wind speed (0, 2, 4, 6 m/s), vibration frequency (0, 10, 20, 30 Hz). Single-factor and multi-factor coupled tests were conducted, with static conditions (20 °C, 0 m/s, 0 Hz) as control.

After curing to 3 d, 7 d and 28 d, specimens were wiped dry and placed in the test bench. Specimen mass was measured by weighing at intervals to calculate evaporation amount and rate. The test lasted 14 days. Each environmental working group contained three parallel specimens, and the average evaporation index of the three parallel samples was adopted to draw all evaporation curves. The calculation formulas are as follows:

$$W_{t}=\frac{m_{0}-m_{t}}{S}$$

$$v_{t}=\frac{W_{t}-W_{t-1}}{t-\left(t-1\right)}$$

where $W_{t}$ = cumulative evaporation at time *t*, kg/m^2^; $m_{0}$ = initial mass of the specimen, kg; $m_{t}$ = specimen mass at time *t*, kg; S = exposed surface area of the specimen, m^2^; $v_{t}$ = evaporation rate at time *t*, kg/(m^2^·h).

### 2.3. Microscopic Characterization and Mechanical Test Methods

Cement paste samples of C30 and C50 were cured for 7 d and 28 d under static and dynamic conditions (30 °C, 4 m/s, 20 Hz). Multiple microscopic and mechanical tests were performed as follows.

(1) SEM observation

Scanning electron microscopy (accelerating voltage 15 kV) was used to observe hydration product morphology. Samples were ground, polished and gold-sprayed before imaging at different magnifications to identify hydration products and pore features. Three parallel microscopic samples were prepared for each group of static/dynamic working conditions, and representative micrographs were selected after multi-region scanning of each sample.

(2) MIP test

Mercury intrusion porosimetry was applied to characterize pore structure. After 28 d curing, samples were taken from the surface (0–5 mm) and interior (20–25 mm), crushed to 3–5 mm and vacuum-dried for 24 h. Tests were performed on an AutoPore V 9620 instrument (pressure 0.16–1000 psi, pore size 3.2 nm–360 μm) to analyze total porosity, pore size distribution and average pore diameter. Three parallel core samples were extracted from the surface and inner part of concrete for each condition, and the average pore parameters were calculated as the final results.

(3) Hydration degree test

Backscattered electron (BSE) imaging and thermogravimetric analysis (TGA) were used to determine the degree of hydration (DoH).

BSE tests were run under low vacuum (0.1–60 Pa) at 15 kV, 1000× magnification. Thirty images per sample were analyzed by Image-Pro Plus to quantify unhydrated clinker content.

TGA was conducted at a heating rate of 10 °C/min from 25 to 800 °C. The hydration degree was calculated from calcium hydroxide decomposition mass loss in the 400–550 °C range. Lower unhydrated clinker and higher calcium hydroxide indicate a higher hydration degree. Three parallel test pieces were taken for TGA and BSE analysis under each environment, and the average hydration degree was recorded.

(4) Mechanical property tests

Cubic compressive strength, prismatic compressive strength, four-point flexural strength and elastic modulus were tested according to the Test Code for Cement and Concrete of Highway Engineering (JTG E30-2005) [14].

At target ages (7 d, 28 d, 60 d, 90 d), specimens were wiped dry and surface defects were removed. Three parallel specimens were tested for each group of environmental conditions. The arithmetic mean value of qualified specimens was taken as the final mechanical index; specimens with test deviation over 10% were discarded.

Cubic compressive strength was tested on 100 mm cubes at a loading rate of 0.5 MPa/s. Prismatic compressive strength and elastic modulus were tested on 100 mm × 100 mm × 400 mm prisms at 0.5 MPa/s. For elastic modulus, extensometers were mounted symmetrically; preloading to 40% of failure load was repeated 3 times before formal graded loading.

Four-point flexural strength was tested on prisms with a 300 mm span and loading points at the 1/3 span positions, at a loading rate of 0.05 MPa/s.

The strength reduction rate was calculated to quantify mechanical degradation under dynamic evaporation. Standard deviation error bars were added to all mechanical curves.

### 2.4. Statistical Analysis of Experimental Data

All data were processed, fitted and statistically analyzed using Origin 2021, with a unified procedure for evaporation, pore parameters, hydration degree and mechanical properties. Each working condition was equipped with 3 parallel samples, and all analysis results were based on the average of valid parallel specimens. Outliers beyond ±10% range were excluded first. Arithmetic mean, standard deviation and standard error were calculated for each group. Error bars were added to mechanical curves to show dispersion. One-way ANOVA was used to evaluate the significance of temperature, wind speed, vibration frequency and strength grade on evaporation, pore connectivity, hydration and mechanical properties. Significance was defined as *p* < 0.05 (significant) and *p* < 0.01 (extremely significant). The evaporation prediction model was built by multivariate nonlinear regression. Fitting performance was evaluated by coefficient of determination R^2^, mean relative error and coefficient of variation. A ±10% prediction band and 90% confidence interval were used to quantify prediction uncertainty.

## 3. Results

### 3.1. Evaporation Evolution Under Single-Factor Conditions

**Figure 2:**
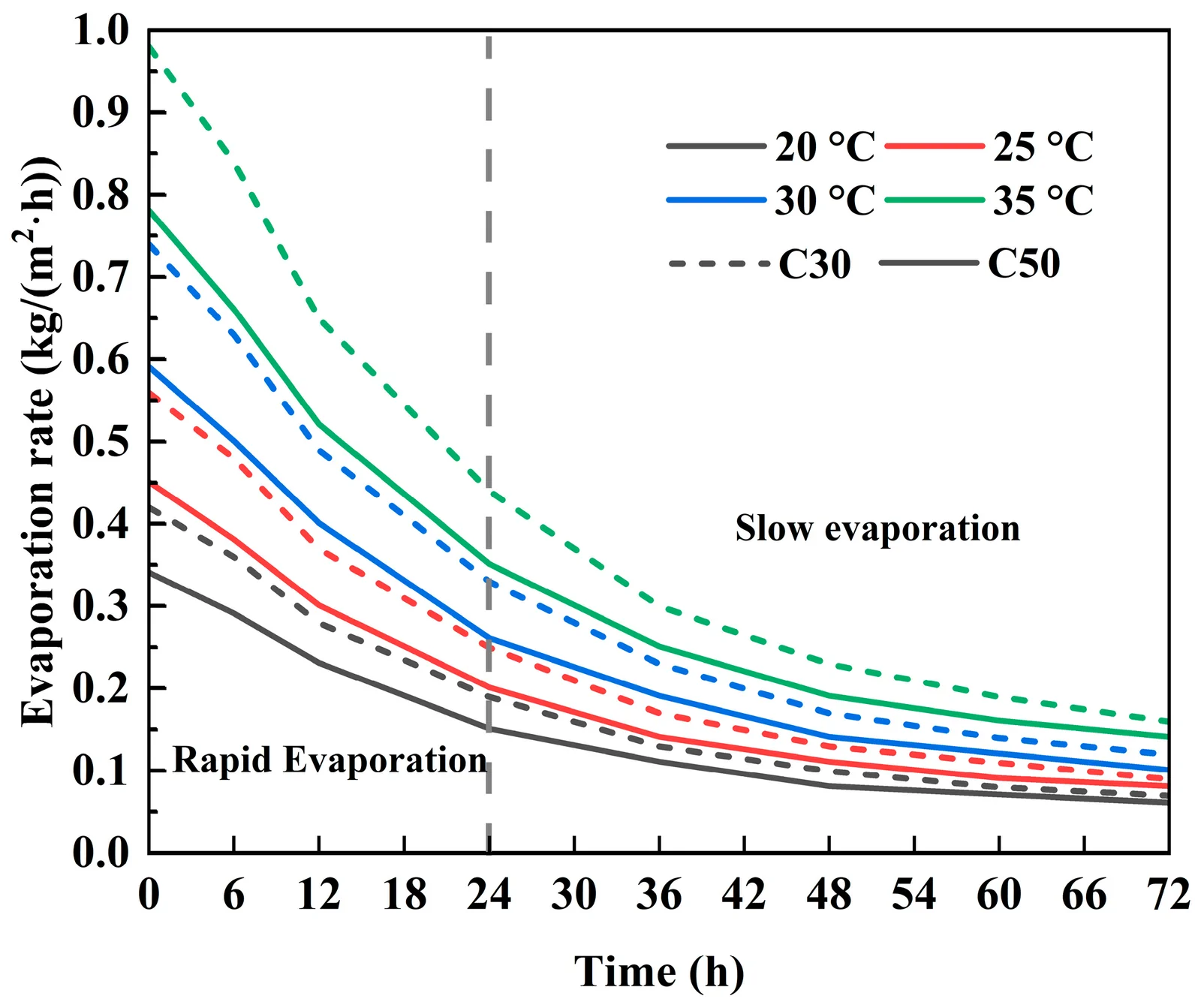
Evaporation rate curves of C30 and C50 concrete at different temperatures.

Temperature is the dominant factor controlling concrete evaporation (Figure 2). The evaporation rate rises markedly with temperature, and the early rate (0–72 h) is much higher than the later stage, showing a fast-to-slow trend. From 20 to 35 °C, each 5 °C increase raises the 72 h average evaporation rate by 32.5% for C30 and 30.2% for C50.

Temperature promotes evaporation in two ways. It accelerates surface water vaporization and enhances gaseous diffusion driving force. It also enlarges humidity and temperature gradients between interior and surface, speeding up inward moisture migration. C50 shows a smaller rate increase because its lower water/binder ratio creates a denser structure and higher migration resistance.

Wind speed affects evaporation by thinning the gaseous boundary layer on the concrete surface. At 0 m/s, a stable boundary layer forms and evaporation is slow. As wind speed increases, the boundary layer breaks down and vapor is carried away rapidly, increasing cumulative evaporation.

**Figure 3:**
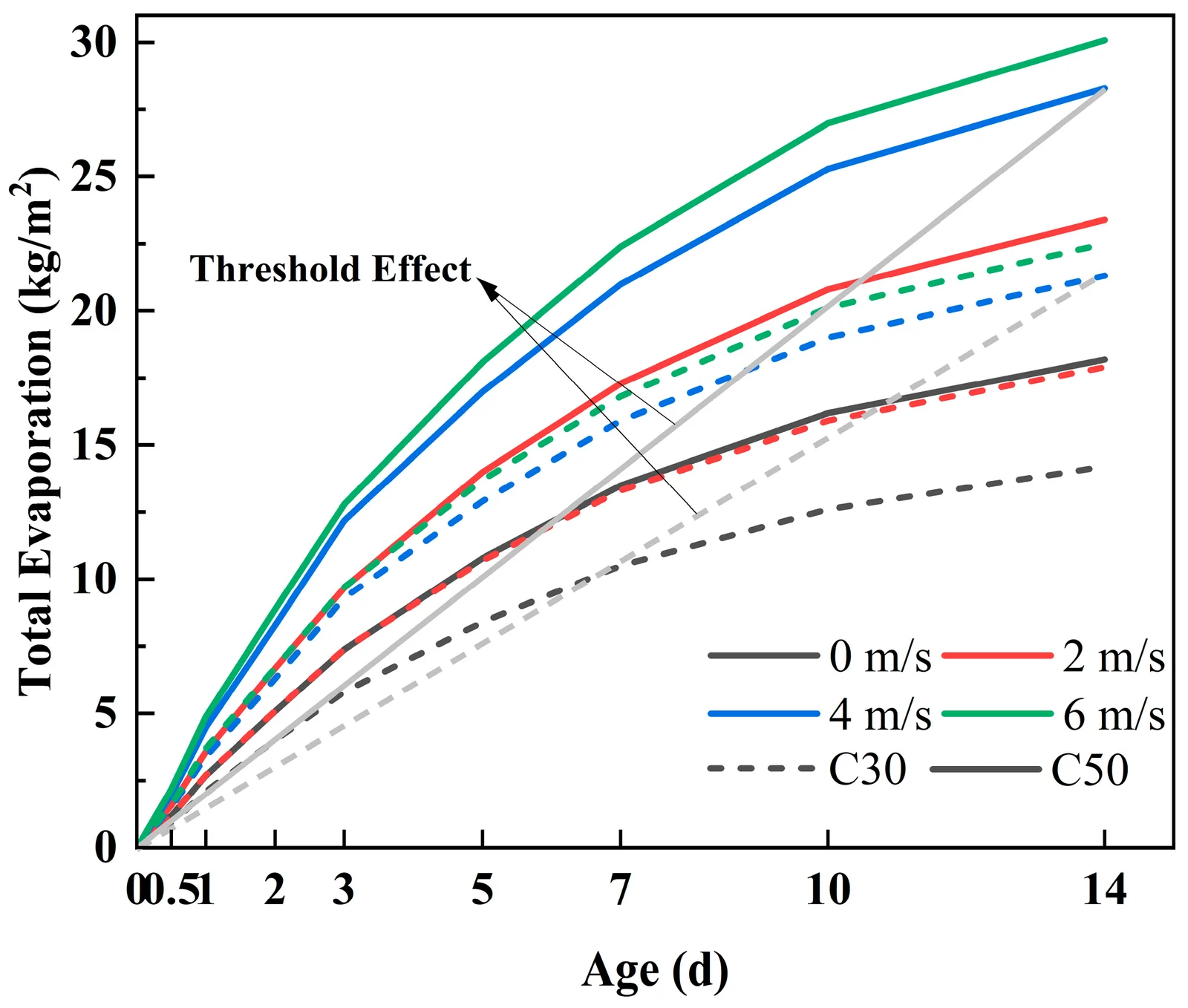
Cumulative evaporation of concrete at different wind speeds.

From 0 to 6 m/s, 14-day cumulative evaporation rises by 65.3% for C30 and 58.7% for C50 (Figure 3). Each 2 m/s wind speed increase raises the early evaporation rate by 28.7% on average. A threshold effect exists around 4 m/s: beyond this value, the boundary layer is fully destroyed, and evaporation is controlled by internal moisture migration rather than external airflow.

**Figure 4:**
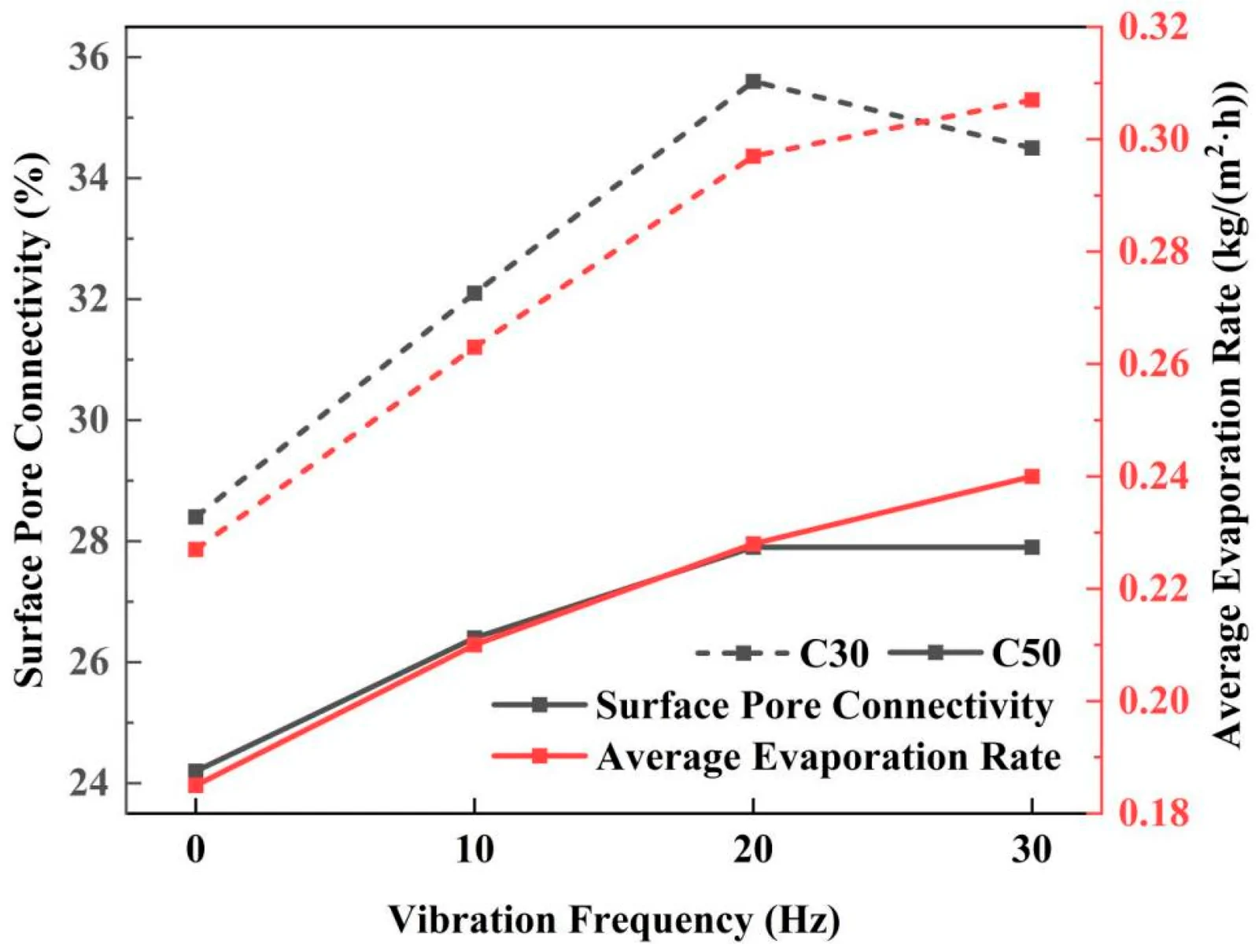
Pore connectivity and evaporation rate at different vibration frequencies.

Traffic vibration alters concrete microstructure and changes evaporation behavior. From 0 to 30 Hz, surface pore connectivity increases by 21.5% for C30 and 15.3% for C50, with corresponding evaporation rate increases of 35.2% and 29.8% (Figure 4).

Vibration rearranges unhardened hydration particles, converting closed surface pores into connected ones and reducing migration resistance. It also generates microcracks that act as fast evaporation channels. C50 has denser hydration products and stronger inter-particle bonding, so vibration causes smaller pore connectivity improvement and lower evaporation enhancement.

### 3.2. Microstructural Changes Under Dynamic Evaporation

**Figure 5:**
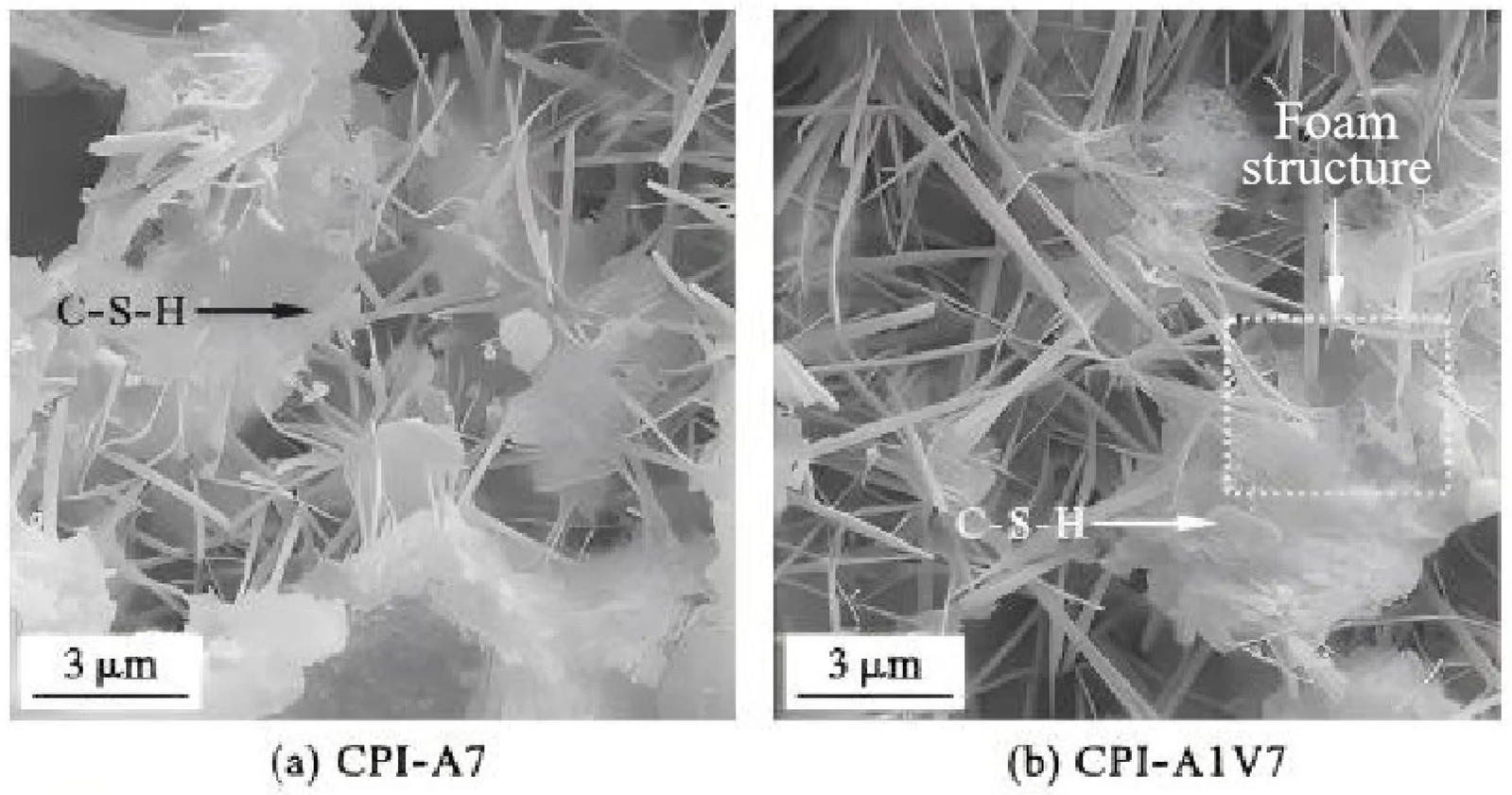
SEM images of concrete under dynamic urban road evaporation. (**a**) CPI-A7 specimen, showing the morphology of C-S-H gel under static environment; (**b**) CPI-A1V7 specimen, displaying C-S-H gel and foam-like pore structure formed under coupled dynamic environment.

SEM observation shows that under static conditions (Figure 5), hydration products are mainly C-S-H gel, calcium hydroxide and ettringite. Dense flocculent C-S-H fills aggregate gaps, with mostly closed pores. Under dynamic conditions, rapid evaporation leads to insufficient hydration: C-S-H appears loose and porous, calcium hydroxide crystals are underdeveloped, ettringite decreases, and connected pores and microcracks increase.

Rapid surface moisture loss prevents full contact between cement particles and water, hindering hydration. Reduced and unevenly distributed hydration products result in a loose microstructure, which accounts for low surface strength and easy cracking of road concrete.

The MIP results show that the dynamic environment significantly increases total porosity and average pore diameter, with much greater changes on the surface than in the interior. Surface total porosity rises from 18.5% to 25.3% for C30 (+36.8%), and from 12.3% to 16.7% for C50 (+35.8%) (Table 3).

Regarding pore size distribution, harmless pores (<20 nm) decrease under dynamic conditions, while harmful pores (20–100 nm) and highly harmful pores (>100 nm) increase markedly, showing a pore coarsening trend (Table 3). This is because rapid evaporation leaves insufficient hydration products to fill pores, and capillary tension during migration expands existing pores.

### 3.3. Hydration Degree Changes Under Dynamic Evaporation

**Figure 6:**
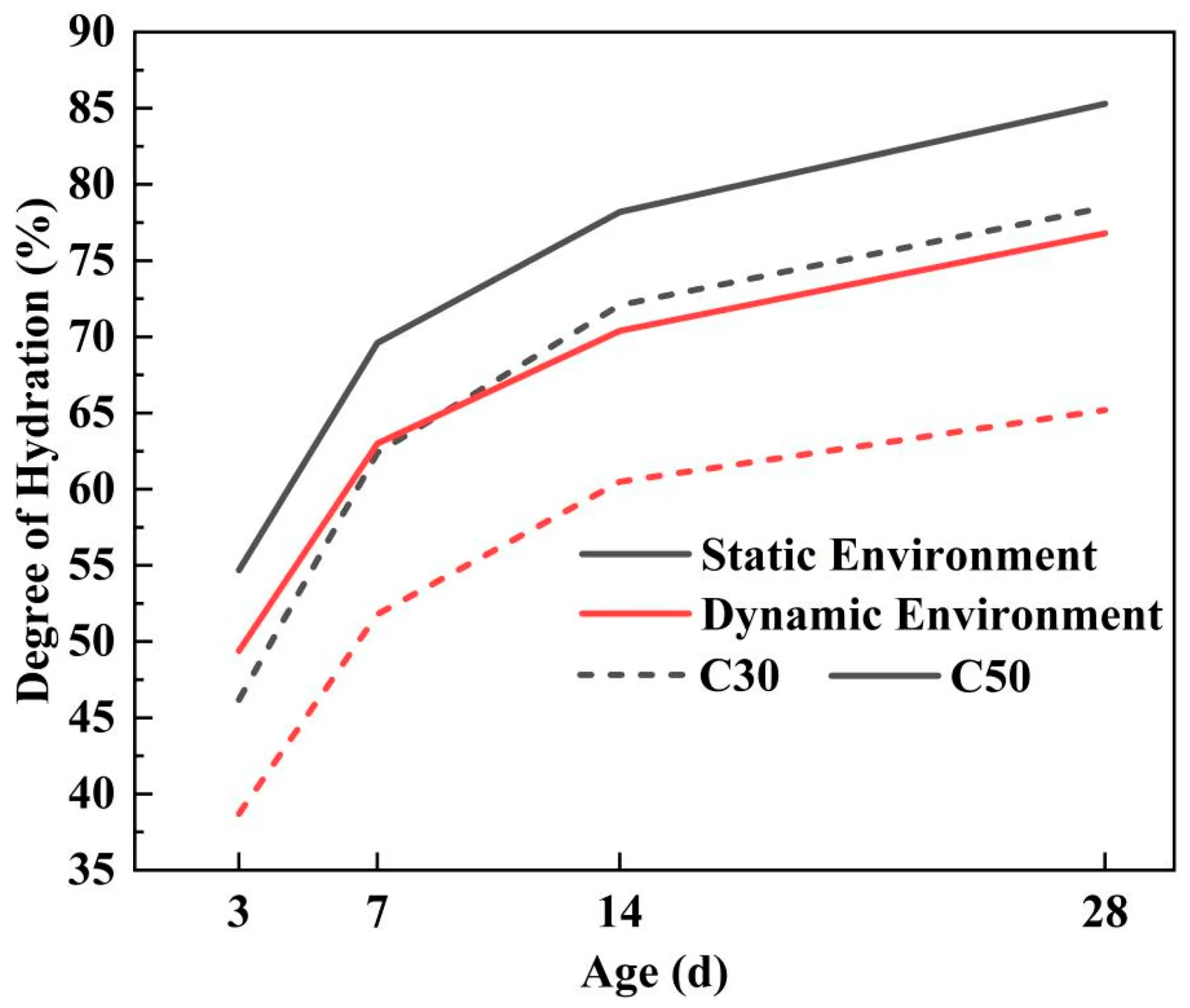
Hydration degree changes under static and dynamic environments.

The TGA results show that at 28 d, the static hydration degree is 78.5% for C30 and 85.3% for C50. Under dynamic conditions, they drop to 65.2% and 76.8%, corresponding to reductions of 16.9% and 9.9% (Figure 6).

Rapid evaporation is the main cause of lower hydration degree. Surface moisture loss starves the hydration reaction and increases unhydrated clinker. The humidity gradient also drives internal moisture toward the surface, affecting inner hydration to some extent.

C50 shows a smaller hydration drop. Its low water/binder ratio means less initial free water, so evaporation has a relatively weaker impact. The secondary hydration of slag powder also partially compensates for hydration loss.

## 4. Discussion

### 4.1. Coupled Evaporation Mechanism Under Dynamic Urban Road Environments

**Figure 7:**
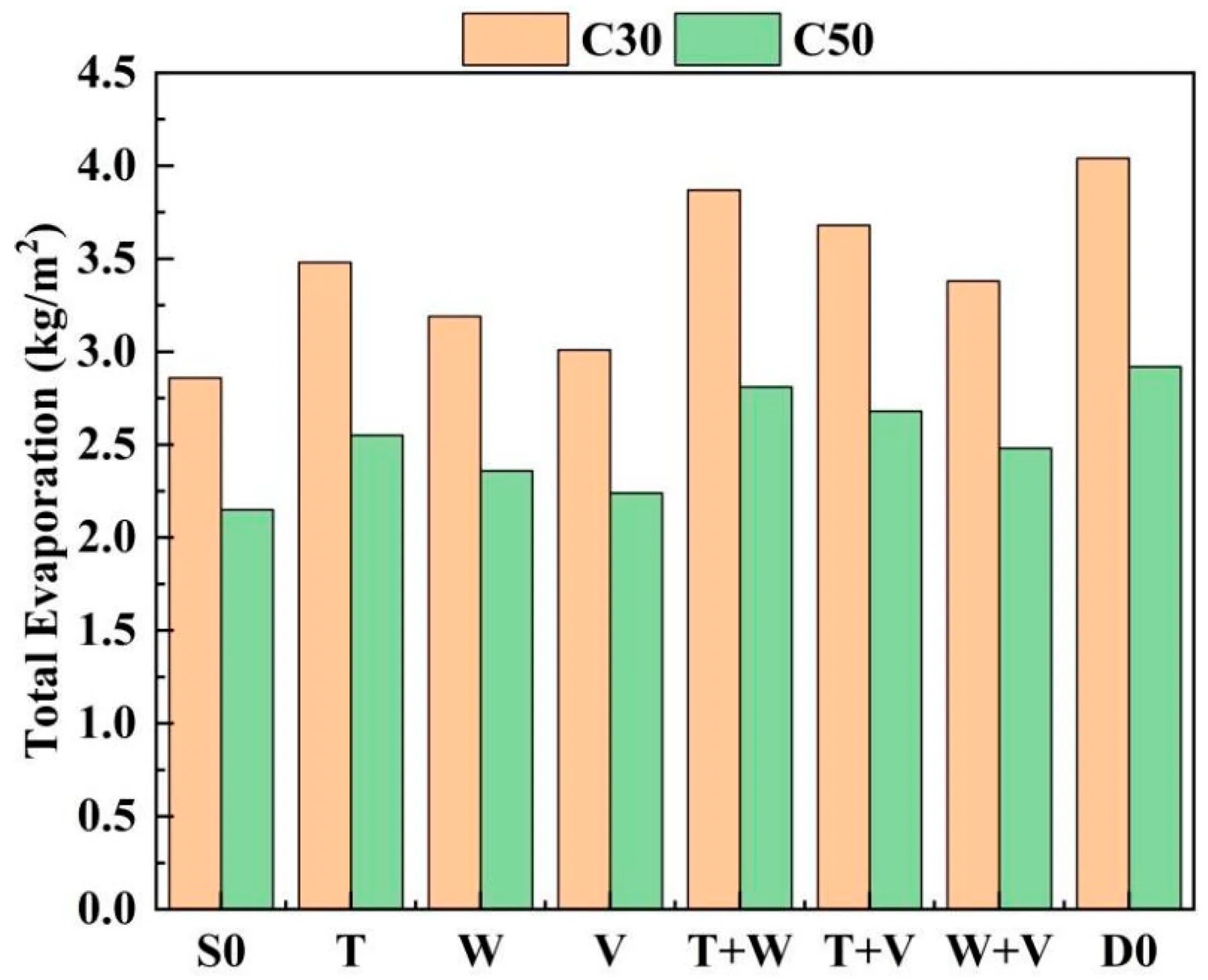
Comparison of evaporation under different combined conditions. Note: S0 = 20 °C + 0 m/s + 0 Hz; T = 30 °C + 0 m/s + 0 Hz; W = 20 °C + 4 m/s + 0 Hz; V = 20 °C + 0 m/s + 20 Hz; T + W = 30 °C + 4 m/s + 0 Hz; T + V = 30 °C + 0 m/s + 20 Hz; W + V = 20 °C + 4 m/s + 20 Hz; D0 = 30 °C + 4 m/s + 20 Hz.

In real urban road service, temperature, wind speed and vibration act together. Under static conditions (20 °C, 0 m/s, 0 Hz), 14-day cumulative evaporation is 2.86 kg/m^2^ for C30 and 2.15 kg/m^2^ for C50. Under coupled dynamic conditions (30 °C, 4 m/s, 20 Hz), it rises to 4.04 kg/m^2^ and 2.92 kg/m^2^, increasing by 41.2% and 35.8% (Figure 7).

The evaporation promotion from multi-factor coupling is not a simple superposition, but a synergistic effect. Orthogonal analysis shows the influence order: temperature > wind speed > load vibration.

Combining single-factor and coupling results, evaporation under dynamic road environments can be divided into three stages: surface vaporization, internal migration and environmental diffusion. They are jointly regulated by temperature, wind speed and vibration, forming a synergistic mechanism: temperature drives vaporization, vibration facilitates migration, wind speed accelerates diffusion.

The surface vaporization stage is dominated by temperature. Higher temperature increases water molecular kinetic energy, speeds up liquid-to-gas conversion, and enlarges the saturated vapor pressure difference between surface and environment.

The internal migration stage is the core link. Load vibration distinguishes road scenarios from static building environments. Vibration rearranges early unhardened particles, opens closed pores and creates microcrack channels, greatly reducing migration resistance.

The environmental diffusion stage is controlled by wind speed. Wind breaks the gaseous boundary layer to speed up vapor removal, but a threshold exists around 4 m/s. Beyond this threshold, evaporation is limited by internal migration, and the marginal effect of wind speed declines.

Under the three-factor coupling, evaporation shifts from single surface diffusion control (static) to joint control by internal migration and surface diffusion. Total evaporation and rate are both higher than simple single-factor superposition, which explains the synergistic effect.

### 4.2. Mechanical Property Evolution Under Dynamic Evaporation

**Figure 8:**
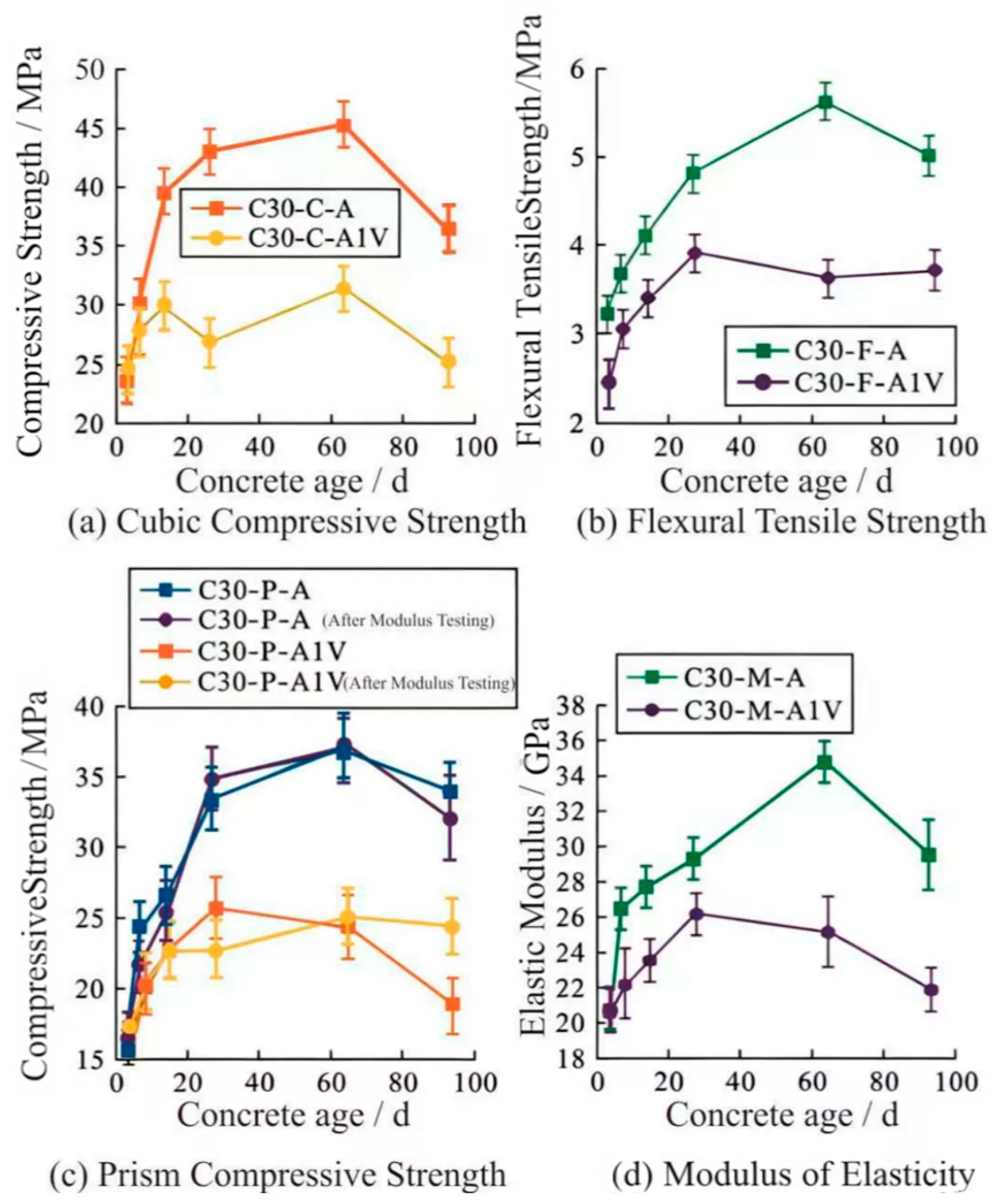
Mechanical properties of C30 concrete with age under static and dynamic environments: (**a**) cubic compressive strength; (**b**) flexural strength; (**c**) prismatic compressive strength; (**d**) elastic modulus.

Microstructural degradation eventually leads to macroscopic mechanical attenuation. Comparing cubic strength, flexural strength, prismatic strength and elastic modulus of C30 and C50 at different ages clarifies the actual impact on pavement bearing capacity (Figure 8).

**Figure 9:**
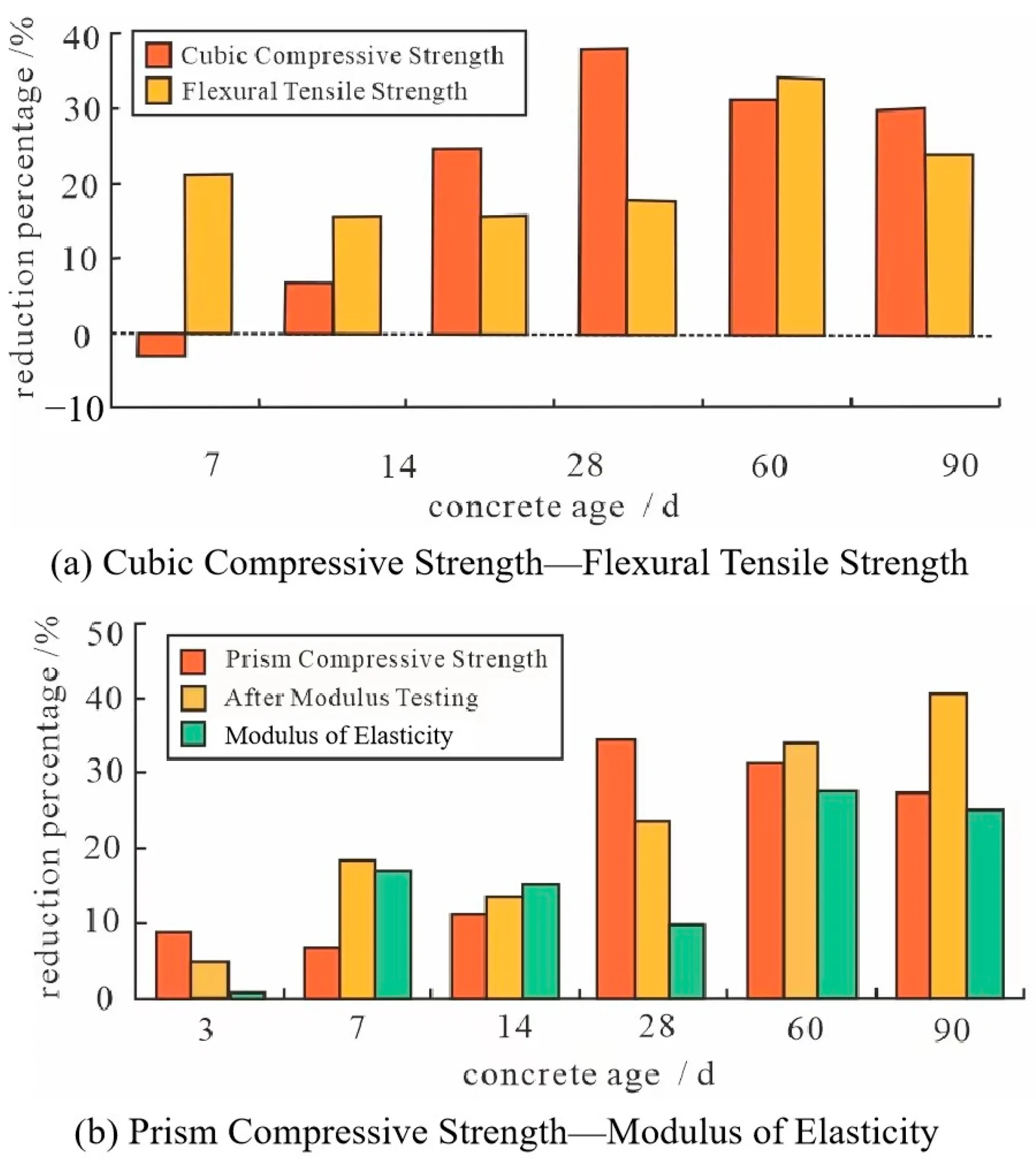
Mechanical reduction rate of C30 concrete with age under dynamic environment. (**a**) Cubic and flexural strength reduction; (**b**) prismatic strength and elastic modulus reduction.

For C30 under static conditions, all mechanical indexes rise with age, peak around 60 d and then stabilize. Under dynamic conditions, indexes are consistently lower, with stage-dependent degradation: small reduction (~2%) at 7 d, peak reduction (~38%) at 28 d, and slight recovery (~30% remaining reduction) at 90 d (Figure 9).

**Figure 10:**
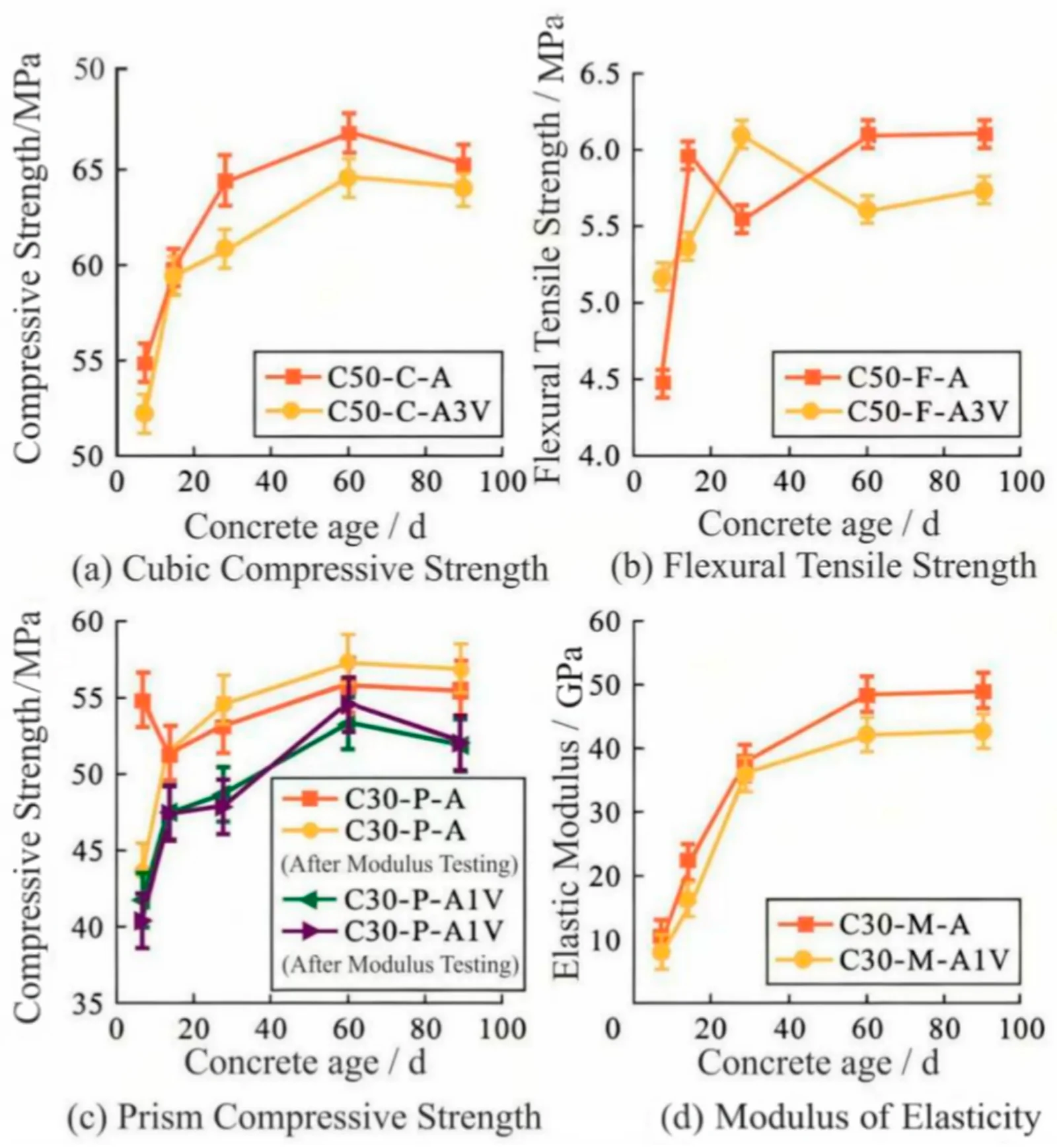
Mechanical properties of C50 concrete with age under static and dynamic environments. (**a**) Cubic compressive strength; (**b**) flexural strength; (**c**) prismatic compressive strength; (**d**) elastic modulus.

Different properties show different sensitivities (Figure 10). Flexural strength responds earlier than compressive strength, with a 22% reduction at 7 d. This is because flexural performance is more sensitive to surface compactness; pore coarsening and microcracks directly weaken bending resistance.

Elastic modulus degradation grows continuously with age, exceeding 25% at 90 d. This indicates dynamic evaporation not only reduces strength but also worsens deformation performance, which is unfavorable for long-term pavement structural behavior.

Under temperature/vibration coupling, vibration amplifies strength loss by 15–25% on average. Higher temperature also intensifies mechanical degradation. This trend matches the evaporation rate pattern well, confirming the chain: temperature → evaporation → micro-damage → mechanical attenuation.

For C50, mechanical degradation is much smaller. The maximum cubic strength reduction appears at 28 d, only 5.3%. Notably, C50 flexural strength at 7 d is slightly higher under dynamic than static conditions. Early vibration compacts low water/binder concrete by optimizing particle packing, partially offsetting evaporation damage. As hydration deficit accumulates after 28 d, flexural strength turns downward but stays within 15% reduction.

Temperature/vibration coupling causes about half the degradation in C50 compared with C30. This demonstrates that low water/binder concrete resists dynamic evaporation better, consistent with its denser pore structure and smaller hydration drop.

**Figure 11:**
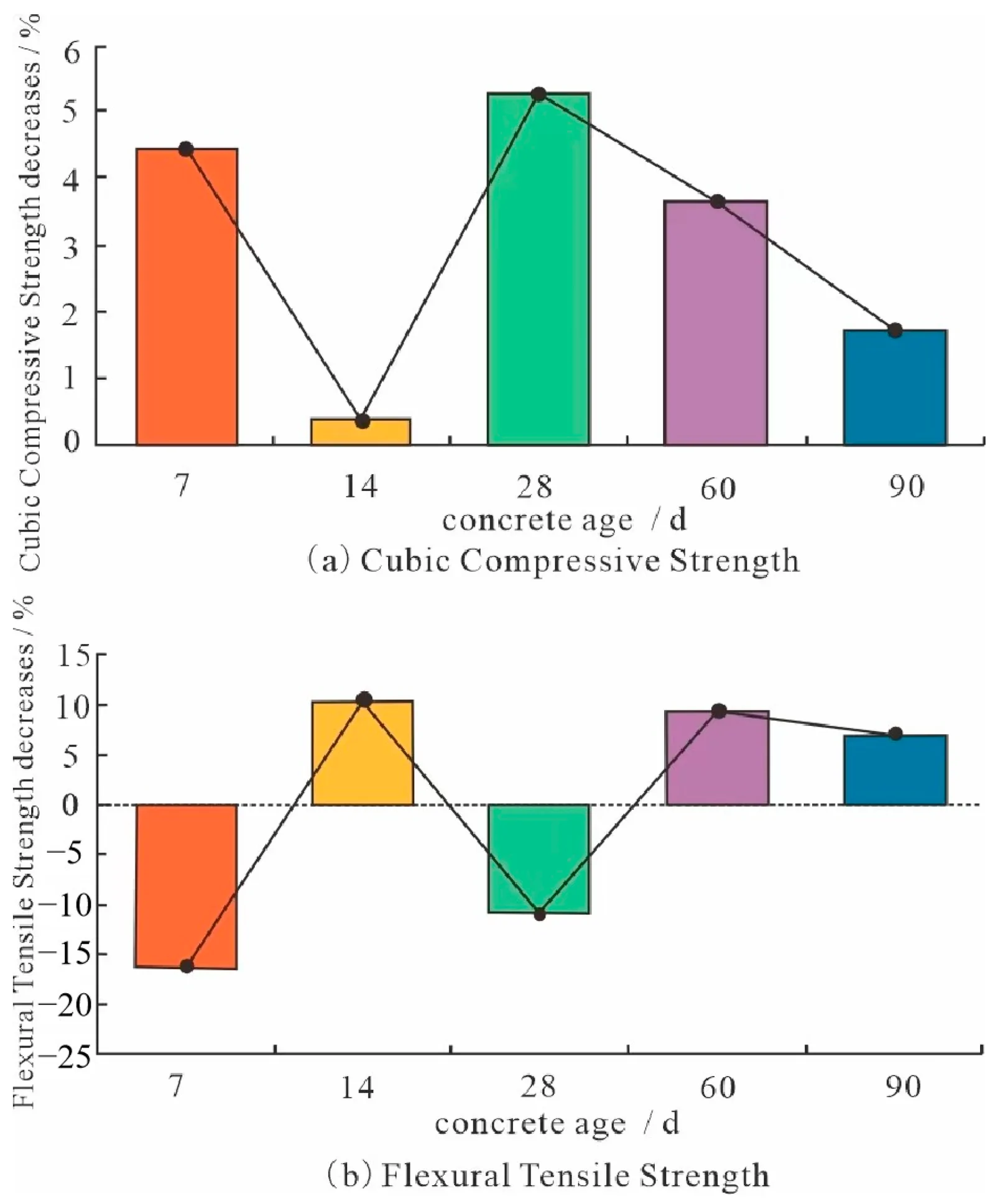
Mechanical reduction rate of C50 concrete with age under dynamic environment. (**a**) Cubic strength reduction; (**b**) flexural strength reduction.

The special strength pattern—slight fluctuation at 7 d, peak degradation at 28 d, mild rebound at 90 d—arises from two competing mechanisms: short-term vibration compaction benefit and long-term evaporation-induced micro-damage (Figure 11).

At 7 d (plastic stage), vibration improves particle packing and compactness. For dense C50, this positive effect dominates, so strength slightly exceeds static samples. For porous C30, vibration accelerates surface water loss and mildly inhibits early hydration, causing a small strength drop.

At 28 d, sustained evaporation creates long-term surface water deficit. C30 surface porosity rises by 36.8% and hydration drops by 16.9%. Irreversible pore coarsening fully cancels early compaction benefit, leading to peak strength loss of nearly 38%. C50 suffers much less damage, namely, 9.9% hydration reduction and 5.3% maximum strength loss, thanks to low water/binder ratio and slag secondary hydration.

At 60–90 d, residual internal moisture redistributes and ongoing slag hydration forms more C-S-H gel, partially refilling pores and recovering strength slightly. However, surface pore coarsening and hydration deficit are permanent. C30 strength at 90 d remains ~30% lower than static. This pattern agrees well with pore structure and hydration test data.

In general, mechanical degradation from dynamic evaporation is the macroscopic expression of microstructural changes, and its magnitude depends on material composition. For heavy-traffic urban road sections, using low water/binder high-strength concrete combined with early moisture curing can effectively mitigate performance loss and extend service life.

### 4.3. Evaporation Prediction Model for Dynamic Urban Road Environments

Based on test data, a cumulative evaporation (*W*) prediction model was established by multivariate nonlinear regression, incorporating temperature (*T*), wind speed (*v*), vibration frequency (*f*), age (*t*) and water/binder ratio (*w*/*b*):

$$W=a\cdot T^{b}\cdot v^{c}\cdot f^{d}\cdot t^{e}\cdot {\left(w/b\right)}^{g}+\;h$$

where *a*, *b*, *c*, *d*, *e*, *g*, and *h* are regression coefficients fitted from the test data.

Least-squares fitting gives: *a* = 0.0025, *b* = 0.852, *c* = 0.426, *d* = 0.215, *e* = 0.368, *g* = 1.523, *h* = 0.125. The final model is

$$W=0.0025\cdot T^{0.852}\cdot v^{0.426}\cdot f^{0.215}\cdot t^{0.368}\cdot {\left(w/b\right)}^{1.523}+0.125$$

Units: *W* in kg/m^2^; *T* in °C; *v* in m/s; *f* in Hz; *t* in d.

**Figure 12:**
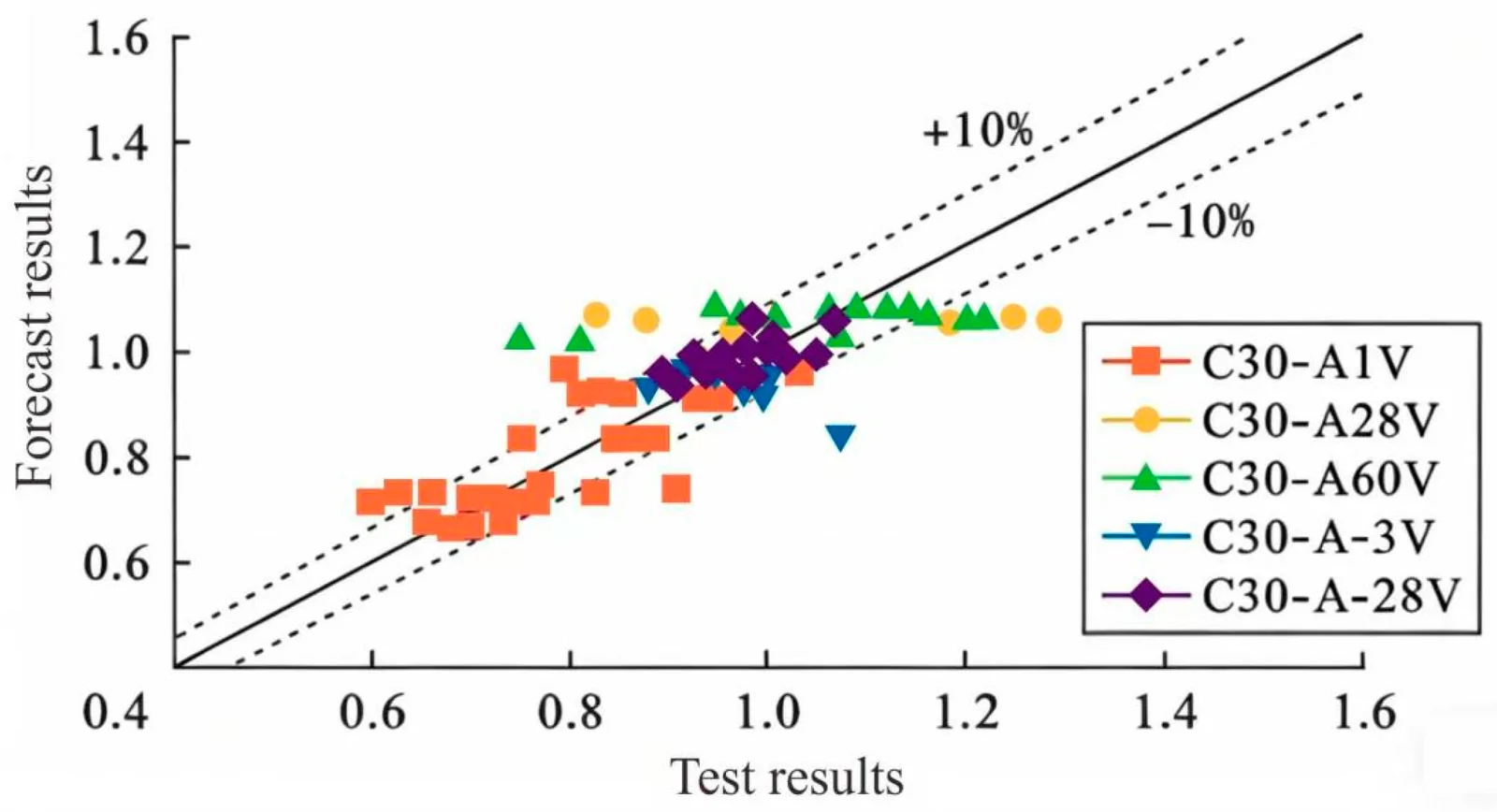
Comparison between predicted and tested values.

Model verification shows an average relative error of 6.5% and a coefficient of variation of 7.8%, indicating good accuracy and reliability (Figure 12). The model applies to temperature −10 to 60 °C, wind speed 0 to 10 m/s, vibration 0 to 50 Hz, age 3 to 28 d, and water/binder ratio 0.40 to 0.55, covering most urban road conditions.

Fitting deviation exists for some groups. At 3–7 d (A1V), abundant free water and active multi-factor driving make evaporation grow nearly linearly, matching the power function form well. Hence A1V has very small error. At 28 d and 60 d, movable free water is largely consumed and capillary channels gradually close. The evaporation rate approaches zero and the cumulative curve flattens. The age term t follows power law growth and cannot precisely capture this late-stage plateau, causing larger deviation for A28V and A60V.

Short-term vibration (A1V) only opens pores and enhances evaporation. Long-term vibration (A28V/A60V) promotes hydration product deposition and reblocks some capillaries, weakening the evaporation gain. The fixed power coefficient for vibration frequency does not account for this time-dependent attenuation, which also contributes to long-age deviation.

The model performs best for 3–14 d early evaporation. Reduced accuracy at 28 d and beyond is a normal boundary limitation.

**Figure 13:**
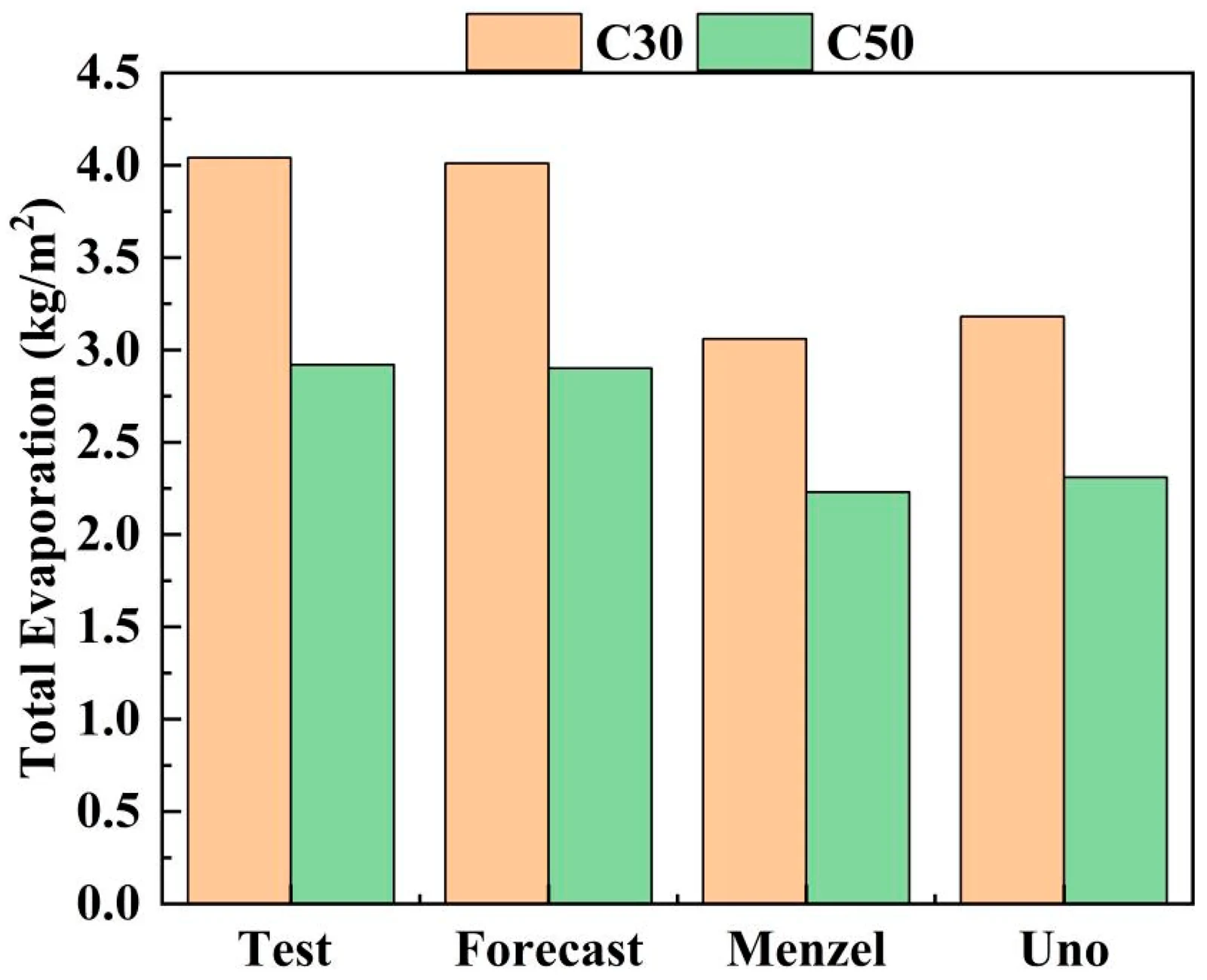
Model accuracy comparison.

To quantify the model advantage, the Menzel [15] and Uno [16] models were selected for comparison under typical dynamic conditions (30 °C, 4 m/s, 20 Hz) (Figure 13).

Both classical models were developed for static open-air casting. They only include temperature, humidity and wind speed, excluding road-specific vibration and water/binder ratio. They cannot reflect vibration-induced pore connectivity improvement or material-dependent migration resistance.

For C30, Menzel gives 3.06 kg/m^2^ (24.3% error) and Uno gives 3.18 kg/m^2^ (21.3% error). Both significantly underestimate dynamic evaporation. The proposed model gives 4.01 kg/m^2^ (0.74% error), improving accuracy by over 20 percentage points.

For C50, Menzel gives 2.23 kg/m^2^ (23.6% error) and Uno gives 2.31 kg/m^2^ (20.9% error). The proposed model gives 2.90 kg/m^2^ (0.68% error), showing similar superiority.

The core error source of classical methods is the mismatch between their static assumption and real dynamic road environments. They ignore vibration-induced pore restructuring and the inhibitory effect of dense low-water/binder matrix. By introducing vibration frequency and water/binder ratio, the proposed model captures the synergistic temperature/wind vibration mechanism and material properties, achieving much higher reliability and engineering applicability for urban road scenarios.

## 5. Conclusions

(1) The evaporation rules and synergistic mechanism under temperature–wind vibration coupling are clarified. Temperature, wind speed and traffic vibration all promote early evaporation with a synergistic effect, in the order of influence: temperature > wind speed > load vibration. A 5 °C temperature rise and 2 m/s wind speed increase raise the early evaporation rate by 32.5% and 28.7% respectively; 30 Hz vibration improves surface pore connectivity by 15.3–21.5%. The three-stage mechanism—temperature drives vaporization, vibration facilitates migration, wind speed accelerates diffusion—shifts evaporation control from single surface diffusion to joint internal-surface control. Dense low-water/binder C50 shows a 14-day evaporation increase that is 5.4 percentage points lower than C30, with better evaporation resistance.
(2) The link between dynamic evaporation, microstructural evolution and mechanical degradation is revealed. Sustained rapid evaporation causes surface water deficit, inhibiting hydration and coarsening pores. At 28 d, hydration degree drops by 16.9% (C30) and 9.9% (C50); surface total porosity rises by 36.8% and 35.8%. The strength pattern—early fluctuation, 28 d peak degradation, late mild rebound—results from competition between short-term vibration compaction benefit and long-term evaporation damage. Low water/binder concrete effectively alleviates mechanical attenuation from dynamic evaporation.
(3) A multi-factor coupled evaporation prediction model is established and validated. The model includes temperature, wind speed, vibration frequency, age and water/binder ratio, with 6.5% average relative error and 7.8% coefficient of variation. It performs best for 3–14 d early evaporation, and improves accuracy by over 20 percentage points compared with Menzel and Uno classical models under dynamic conditions. The model covers mainstream urban road environments and concrete mixes, and can provide quantitative support for mix design, curing strategy and durability improvement.

## Figures and Tables

**Table 1:** Chemical composition of P·I 42.5 cement (mass fraction/%).

SiO_2_	Al_2_O_3_	Fe_2_O_3_	CaO	MgO	SO_3_	Na_2_O	K_2_O	Cl^−^
20.60	4.57	3.29	63.27	2.59	2.11	0.55	0.76	0.026

**Table 2:** Chemical composition of P·I 42.5 cement (%).

Strength Grade	Water/Binder Ratio	Water (kg/m^3^)	Cement (kg/m^3^)	Slag Powder (kg/m^3^)	Medium Sand (kg/m^3^)	Basalt Aggregate (kg/m^3^)
C30	0.55	230	420	0	665	1085
C50	0.4	320	60	893	967	170

**Table 3:** Pore structure indexes under static and dynamic environments.

Concrete Type	Environment	Position	Total Porosity/%	Average Pore Size/nm	Harmless Pores (<20 nm)/%	Harmful Pores (≥20 nm)/%
C30	Static	Surface	18.5	45.2	42.3	57.7
C30	Dynamic	Surface	25.3	78.5	28.6	71.4
C30	Dynamic	Interior	20.1	52.3	38.5	61.5
C50	Static	Surface	12.3	32.5	56.8	43.2
C50	Dynamic	Surface	16.7	55.7	41.2	58.8
C50	Dynamic	Interior	13.5	36.8	52.1	47.9

## Data Availability

The original contributions presented in this study are included in the article. Further inquiries can be directed to the corresponding author.
